# Book Reviews

**Published:** 1896-07

**Authors:** 


### Book Reviews.

A TREATISE ON THE MEDICAL AND SURGICAL DISEASES OF INFANCY AND CHILDREN. By J. Lewis Smith, M. D., Clinical Professor of Diseases of Children, Bellevue Hospital Medical College, Physician to Charity Hospital, etc. Eighth Edition, Thoroughly Revised and Greatly Enlarged. With 273 illustrations and 4 plates. New York and Philadelphia: Lee Bros. & Co., 1896.

A large number of recent and pretentious notes upon the diseases of childhood have recently been published, but none of them have displaced the present work, nor are they likely to do so. No man has written more clearly and concisely upon the diseases of children than J. Lewis Smith, and this eighth edition of his work comes to us after a careful and thorough revision, with portions of the seventh edition rewritten, while some entirely new matter has been added; notably, the Surgical Diseases of Childhood. This portion of the work is contributed by Dr. Stephen Smith. Dr. F. M. Warner has contributed a section on Diseases of the Blood, this being among the last work done by him prior to his death.

A very large number of new illustrations have been added, while the defective old ones were withdrawn. All portions of the work are brought fully up to date, and altogether the eighth edition is notably superior to all former editions and we cannot too heartily commend the author's last effort to the profession.

---

TWENTIETH-CENTURY PRACTICE OF MEDICINE. VOL. VI. Diseases of the Respiratory Organs. New York: Wm. Wood & Co.

According to the publishers, "Owing to unforeseen difficulties in the preparation of Vol. V., it has been found necessary to issue Vol. VI. out of its natural order."

This volume treats of the diseases of the following organs:

The Nose: the Accessory Sinuses of the Nose, Naso-pharynx, Pharynx, Ear, Tonsils, Larynx, Trachea and Bronchial Tubes, Lungs. The men who have been assigned to the treatment of these various subjects are all men of reputation and ability, which is fully exemplified in the manner in which the subjects are treated. The pathology and treatment are thoroughly up to date, and this volume we can recommend as a valuable addition to any physician's library, especially such as do rhinological and laryngological work. The contributors are to be congratulated upon the thoroughness of their work.

